# QuEChERS Method Followed by Solid Phase Extraction Method for Gas Chromatographic-Mass Spectrometric Determination of Polycyclic Aromatic Hydrocarbons in Fish

**DOI:** 10.1155/2015/352610

**Published:** 2015-03-19

**Authors:** Mona Khorshid, Eglal R. Souaya, Ahmed H. Hamzawy, Moustapha N. Mohammed

**Affiliations:** ^1^Central Laboratory of Residue Analysis of Pesticides and Heavy Metals in Food (QCAP), Agricultural Research Center, Ministry of Agriculture and Land Reclamation, 7 Nadi Elsaid Street, Dokki, Giza 12311, Egypt; ^2^Department of Chemistry, Faculty of Science, Ain Shams University, Cairo 11566, Egypt

## Abstract

A gas chromatography equipped with mass spectrometer (GCMS) method was developed and validated for determination of 16 polycyclic aromatic hydrocarbons (PAHs) in fish using modified quick, easy, cheap, effective, rugged, and safe (QuEChERS) method for extraction and solid phase extraction for sample cleanup to remove most of the coextract combined with GCMS for determination of low concentration of selected group of PAHs in homogenized fish samples. PAHs were separated on a GCMS with HP-5ms Ultra Inert GC Column (30 m, 0.25 mm, and 0.25 *µ*m). Mean recovery ranged from 56 to 115%. The extraction efficiency was consistent over the entire range where indeno(1,2,3-cd)pyrene and benzo(g,h,i)perylene showed recovery (65, 69%), respectively, at 2 *µ*g/kg. No significant dispersion of results was observed for the other remaining PAHs and recovery did not differ substantially, and at the lowest and the highest concentrations mean recovery and RSD% showed that most of PAHs were between 70% and 120% with RSD less than 10%. The measurement uncertainty is expressed as expanded uncertainty and in terms of relative standard deviation (at 95% confidence level) is ±12%. This method is suitable for laboratories engaged daily in routine analysis of a large number of samples.

## 1. Introduction

Polycyclic aromatic hydrocarbons (PAHs) are a large group of organic compounds that are included in the European Union (EU) and US Environmental Protection Agency (US EPA) priority pollutant list due to their mutagenic and carcinogenic properties [1]. The most important sources of PAHs have been identified as coke ovens in the production of aluminum, iron, and steel; heating in power plants and residences; cooking; motor vehicle traffic; environmental tobacco smoke; and the incineration of waste material [2]. Cooking and food processing at high temperatures have been shown to generate various kinds of genotoxic substances or cooking toxicants including PAHs [3]. A number of PAHs are known for their carcinogenic, mutagenic, and teratogenic properties like benzo(a)anthracene, benzo(b)fluoranthene, benzo(k)fluoranthene, benzo(g,h,i)perylene, benzo(a)pyrene, chrysene, dibenzo(a,h)anthracene, and indeno(1,2,3-cd)pyrene [4]. PAHs containing up to four fused benzene rings are known as light PAHs and those containing more than four benzene rings are called heavy PAHs. Heavy PAHs are more stable and more toxic than light ones [5]. Light PAHs are more volatile, water soluble, and less lipophilic than the heavy PAHs, so PAHs migrate through the food chain into hydrophobic compartments and thus accumulate in lipid components due to their lipophilic nature [6–8].

Seven of the PAHs have been classified by the US EPA as compounds of probable human carcinogens. These are benzo(a)anthracene, benzo(b)fluoranthene, benzo(k)fluoranthene, chrysene, benzo(a)pyrene, dibenzo(a,h)anthracene, and indeno(1,2,3-cd)pyrene [9]. With the aim of minimizing harmful effects on human health, recently, the European Union established a maximum level of 2 ng/g wet weight for benzo(a)pyrene (the marker used for carcinogenic risk of PAHs) in muscle meat of fish [10].

In 2008, a scientific opinion adopted by the European Food Safety Authority (EFSA, 2008) concluded that benzo(a)pyrene alone is not a suitable indicator for the occurrence and toxicity of PAHs in food and that eight specified PAHs (PAH_8_), for which oral carcinogenicity data are available, and/or a subgroup of these, PAH_4_, are more suitable markers. It was further concluded that PAH_8_ would not provide much added value compared to PAH_4_ (the sum of benzo(a)pyrene, chrysene, benz(a)anthracene, and benzo(b)fluoranthene) [11]. In September 2012, benz(a)anthracene, benzo(b)fluoranthene, and chrysene were included in the assessment and recorded together with benzo(a)pyrene as a sum parameter (group of “PAH_4_”), as per Regulation (EU) number 835/2011.

Developed analytical methods include soxhlet extraction [12], dispersed solid phase extraction [13], and accelerated solvent extraction coupled to sample cleanup using gel permeation chromatography [14] which had been used to assess most of PAHs in different matrices by changing the technique of cleanup from coextracted interferences that may cause false positive results, but most of these techniques are expensive, use chlorinated solvent for extraction, and are time and chemicals consuming. In 2013 a simple solid phase extraction (SPE) method [15] followed by comprehensive two-dimensional gas chromatography coupled to time-of-flight mass spectrometry has been developed for analysis of (15 + 1) carcinogenic polycyclic aromatic hydrocarbons (PAHs). This method includes three critically assessed sample preparation approaches: (i) gel permeation chromatography (GPC), (ii) GPC followed by silica based SPE, and (iii) SPE employing PAHs-dedicated molecularly imprinted polymers (MIPs).

Also in 2013, two of the most relevant analytical methods including different extraction procedures such as ultrasound-assisted solvent extraction (USAE) and ultrasound-assisted emulsification microextraction (USAEME) for determination of 11 mutagenic and carcinogenic PAHs were optimized by the selected extraction techniques. The recoveries ranging from 70% to 100% by USAE and from 70% to 108% by USAEME with estimated quantification limits between 0.020 and 2.6 *μ*g/kg were achieved [16].

A few researches on the development of QuEChERS analytical method for determination of PAHs levels in fish have been previously published in the literature. The streamline of QuEChERS (quick, easy, cheap, effective, rugged, and safe) method for extraction of pesticides in tissues of high fat (>3.5%) encourages scientists to apply modifications and develop this method in order to extract veterinary drugs [17] and PAHs from seafood such as shrimp [18] and in fish by using QuEChERS for extraction followed by dispersive SPE analysis by GCMS in SIM mode for quantification [19].

The aim of this study is to adapt and validate QuEChERS method [20] for extraction followed by solid phase extraction for sample purification and gas chromatography mass spectrometer GCMS for determination of 16 PAHs in fish at low LOQ level.

## 2. Materials and Methods

### 2.1. Sample Preparation

The edible parts (head, bones, and removable skin were removed) of nonsmoked blank Herring fish were obtained and completely homogenized in a food mixer as a blank sample and then stored in a freezer at −20°C.

### 2.2. Chemicals and Reagents

Acetone (Riedel-de Häen, purity 99.8%), acetonitrile (Sigma-Aldrich, purity > 99.9%), toluene (Merck), dichloromethane chromatography grade, and n-hexane (purity > 99.0%) were the solvents used. Agilent QuEChERs salts and buffers were prepackaged in anhydrous packages for EN 15662 containing 4 g magnesium sulfate (MgSO_4_), 1 g sodium chloride (NaCl), 1 g sodium citrate, and 0.5 g disodium citrate sesquihydrate. Silica gel (60–120 mesh, Fluka) was activated at 150°C for 12 hours prior to use.

A 1000 *μ*g/mL stock solution of 14 PAHs includes naphthalene, fluorene, fluoranthene, benz(a)anthracene, chrysene, pyrene, benzo(b)fluoranthene, benzo(k)fluoranthene, benzo(a)pyrene, acenaphthene, phenanthrene, anthracene, acenaphthylene, and pyrene-d_10_ (surrogate standard) and reference standards obtained from Sigma-Aldrich with purity > 95% were prepared, while benzo(g,h,i)perylene and dibenz(a,h)anthracene were obtained as readymade of 100 *μ*g/mL in methylene chloride and indeno[1,2,3-cd]pyrene 200 *μ*g/mL in methanol. A 1 *μ*g/mL working solution of all 16 PAHs was prepared in toluene. Calibration mixtures with concentration 2, 10, 50, 100, and 500 ng/mL were prepared from serial dilution of the working solution in toluene where pyrene-d_10_ maintained at level 50 ng/mL in all calibration levels and all stored in refrigerator at 4°C.

### 2.3. Apparatus

PFTE or polyethylene 50 mL tubes with screw cap and 15 mL tubes contain 1 g magnesium sulfate were obtained for sample extraction. Centrifuge up to 4000 rpm (Heraeus Labofuge 400), Vortex, Automatic Pipettes (Hirschmann Laborgerate) suitable for handling volumes of 10 *μ*L to 100 *μ*L and 100 *μ*L to 1000 *μ*L, 10 mL solvent-dispenser (Hirschmann Laborgerate) for Acetonitrile. The glassware were washed with detergent and water then rinsed with acetone and dried at 90°C before use.

### 2.4. Sample Extraction Steps

The validation procedure needs to be considered, the context of fitness for purpose and cost benefit criteria [21]. About 10 g of fish sample was weighted in 50 mL Teflon centrifuge tube, 50 *μ*L of 10 *μ*g/mL pyrene-d_10_ was added which acts as surrogate standard of 50 *μ*g/Kg, and each set of 6 replicates was spiked with 20, 100, and 500 *μ*L of 1 *μ*g/mL spiking mixture to get 2, 10, and 50 *μ*g/kg, respectively. 10 mL of acetonitrile was used for extraction, shaken for 2 minutes, mixed with Agilent QuEChERs, shaken for 1 minute, and centrifuged at 4000 rpm for 5 minutes. Aliquots of the resulting supernatant were transferred to Teflon tube containing MgSO_4_, vortexed for 30 seconds, and centrifuged at 4000 rpm for 2 minutes; 4 mL of the acetonitrile layer was transferred into 50 mL flask and then evaporated near to dryness.

### 2.5. Cleanup of PAHs Samples by Packed Solid Phase Extraction (SPE) Steps

All fish extracts were subjected to packed solid phase cleanup cartridge which was prepared in-house as follows. Plug a glass wool on 10 mL length syringe; 1 g 20% deactivated silica gel and 0.2 MgSO_4_ were weighted and conditioned with 5 mL of n-hexane/dichloromethane (3 : 2), the sample extract loaded to the cartridge using 10 mL of elute (n-hexane/dichloromethane). Collect fractions in a 50 mL flask, evaporate on rotary evaporator at 40°Cnear to dryness and dissolve in 2 mL toluene and then apply to GCMS for analysis.

### 2.6. GC-MSD Conditions

Agilent 6890N series gas chromatography instrument equipped with 5975 series mass selective detector and Agilent GC Column of model J&W HP-5ms Ultra Inert with the specifications (30 m length, 0.25 mm internal diameter, 0.25 *μ*m film thickness) were used for both qualitative and quantitative determination of PAHs. Helium gas was used as the carrier gas; the column was maintained at a constant flow rate of 1.3 mL/min. The back injector line was maintained at 260°C. Injection volumes were 1.0 *μ*L in the splitless mode. The column temperature was initially held at 90°C for 2 min, ramping to 180°C at a rate of 15°C/min, held at 180°C for 15 min, ramping to 250°C at a rate of 10°C/min, held for 2 min, ramping to 290°C at a rate of 10°C/min, and held for 10 min. The mass spectrometer was operated in the ionization mode and spectra were acquired using a mass range of 45–450 m/z. SIM acquisition was carried out by comparison of the base peak of each targeted PAH as shown in Table 1.

Quality control and assurance of each patch were passed by monitoring the performance of the GCMS and the mass selective detector daily by tuning the mass detector and monitoring the sensitivity and linearity of the calibration curve, respectively, and also analyzing blank sample to confirm that there in contamination effect on the results during analysis.

## 3. Results and Discussion

### 3.1. Chromatographic Results

**Figure 1:**
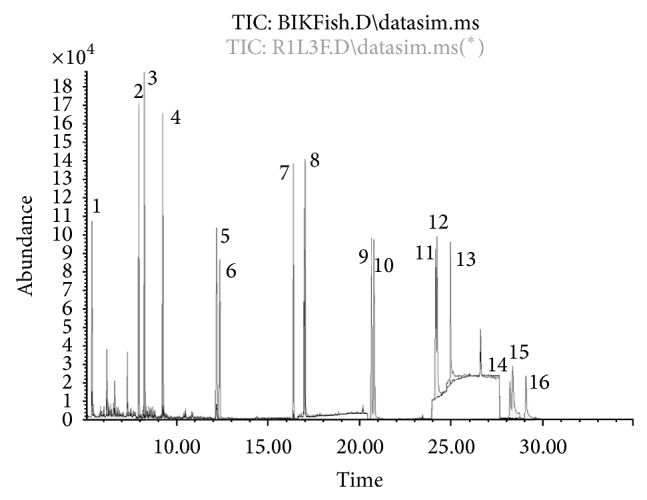
Representing total ion chromatogram of 16 PAHs in fish sample at level 50 *μ*g/kg by weight.

Figure 1 represents overlay between blank and spike fish at level 50 *μ*g/kg samples to show the separation of 16 PAHs by GCMS in 35 minutes using Agilent J&W HP-5ms Ultra Inert GC Column (30 m length, 0.25 mm internal diameter, and 0.25 *μ*m film thicknesses). PAHs corresponding to chromatogram numbers can be found in Table 1. This representative chromatogram of PAHs in fish matrix indicates good cleanup separation techniques with minimum interference of coextract that may influence the accuracy of the result. Matrix matched standards were used in order to compensate the matrix enhancement effect. This indicates good selectivity and specificity of the method.

### 3.2. Method Linearity

The linearity was obtained by plotting the peak area of each analyte versus its concentration.

The linearity of all PAHs indicates that both dibenz(a,h)anthracene and indeno(1,2,3-cd)pyrene compounds had *r* ^2^ values of 0.996; all others were 0.998 or higher within measurement range of 2–50 *μ*g/L indicating excellent linearity.

### 3.3. The Limit of Determination (LOD)

It is the minimum concentration of analyte in the test sample that can be measured with a stated probability that the analyte is present at a concentration above that in the blank sample. Limits of detection expressed as three multiplied by SD of the recovery replicates at the lowest expected concentrations ranging between 0.09 and 1.94 *μ*g/kg are shown in Table 2.

### 3.4. The Limit of Quantitation (LOQ)

It is the lowest concentration of analyte that can be determined with an acceptable level of uncertainty according to Eurachem guideline and it is usually the lowest point on the calibration curve which is 2 *μ*g/kg. The analytes were considered to be quantitative when their abundance confirmation ion signal to noise is S/N ≥ 3 with an accurate quantitation of ±20% of their true value in the calibration standard. Sample residues that met all criteria but had S/N < 3 were reported as less than the limit of quantification (<LOQ) while those which had not fit any criteria were reported as not detected (N.D.).

### 3.5. Recovery and Relative Standard Deviation (RSD)

The recovery of (*n* = 6) replicates at each level was calculated and summarized in Table 3 which shows very good recovery and excellent RSD.

From Table 3, the recovery of each set of 6 replicates was in the range of 56–115% where the lower spiking level was selected in order to include the lower concentration of PAHs fish muscle fixed at 2 *μ*g/Kg. The extraction efficiency was consistent over the entire range with indeno(1,2,3-cd)pyrene and benzo(g,h,i)perylene being the most affected compounds where their recovery at lower level was 65 and 69%, respectively, and at the second level was 61 and 56%. No significant dispersion of results was observed for the other remaining PAHs and recovery did not differ substantially at the lowest and the highest concentrations.

According to Commission Regulation (EC) number 1881/2006 and (EC) number 333/2007 [22, 23], the maximum level for the determination of PAHs in fish was 2 *μ*g/kg wet weight and the recovery range of the methods used should be 50–120%, indicating that the validated method complies with these criteria.

Where RSD_pooled_ can be calculated [21] as

(1) $$\begin{array}{c} {\text{RSD}}_{\text{pooled}}=\sqrt{\frac{{\text{RSD}}_{1}^{2}\left(n_{1}-1\right)+{\text{RSD}}_{2}^{2}\left(n_{2}-1\right)+\cdots }{\left(n_{1}-1\right)+\left(n_{2}-1\right)+\cdots }}. \end{array}$$

RSD is the relative standard deviation, *n* is the number of samples, and the equation used to calculate the recovery is [24]

(2) $$\begin{array}{c} \text{Recovery}\%=\frac{C_{f}}{C_{e}}\times 100, \end{array}$$

where *C*_*f*_ is the found concentration and *C*_*e*_ is the expected concentration.

**Figure 2:**
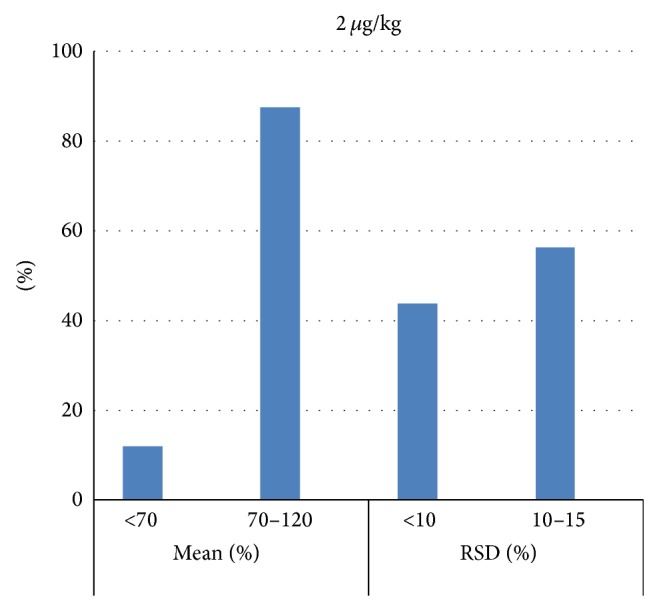


**Figure 3:**
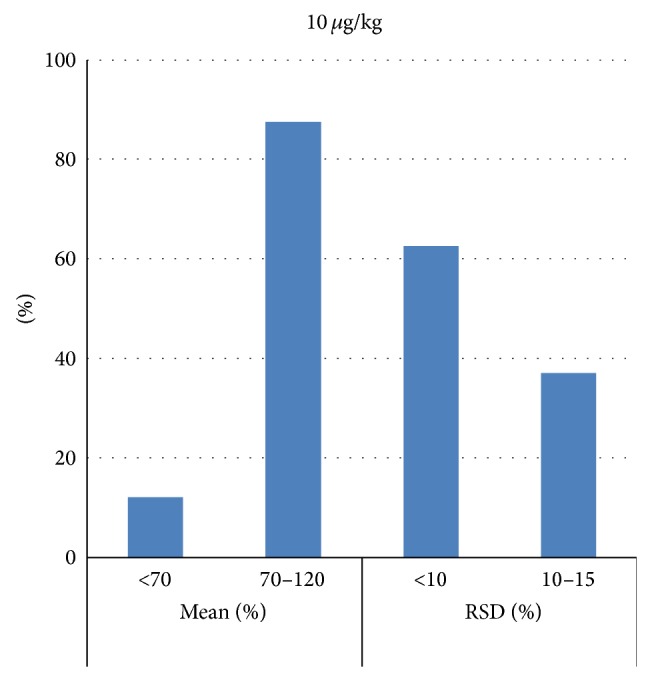


**Figure 4:**
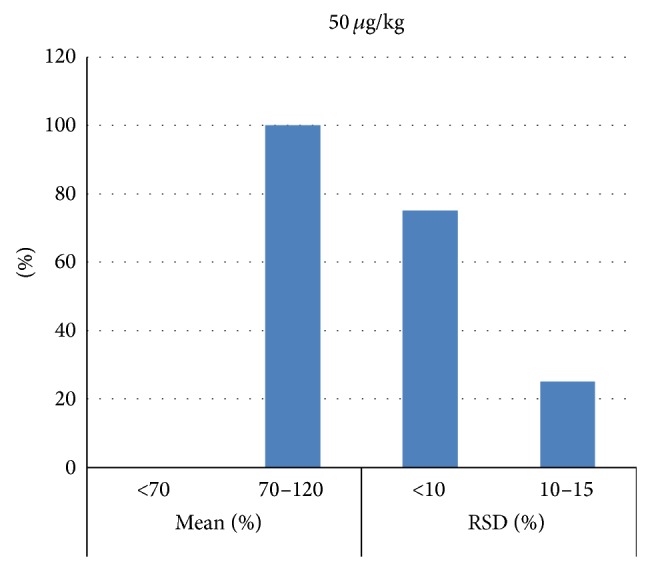


Figures 2, 3, and 4 represent mean recovery and RSD% ranges; most of the PAHs recovery was between 70 and 120% with most of RSD less than 10%.

The reported results provide evidence that the adapted QuEChERS method achieved for most of the PAHs gives good recoveries, repeatability, and reproducibility.

### 3.6. Method Uncertainty Calculation

Using these equations the following was found.

Relative standard uncertainty *U*_Rec_ = 3.6% and

(3) $$\begin{array}{c} U_{\left(\text{Rec}\right)}=\frac{s}{\sqrt{n}}. \end{array}$$

Combined uncertainty *U*_*c*_ is

(4) $$\begin{array}{c} U_{c}=\sqrt{{\left(U_{p}\right)}^{2}+{\left(U_{\text{Rec}}\right)}^{2}+U_{\text{Ref}}}=6.2\%. \end{array}$$

*U*_*c*_ is combined uncertainty. *U*_Rec_ is the uncertainty due to recovery. *U*_Ref_ is the uncertainty due to reference standard preparation. *U*_*p*_ is the uncertainty due to precision experiments.

The uncertainty due to reference standard preparation *U*_Ref_ = 0.7.

*U*_*p*_ which is the relative standard uncertainty due to precision experiments expressed as relative standard deviation was found to be less than 5% (the highest pooled RSD% for pyrene).

Expanded uncertainty is obtained by multiplying the combined uncertainty by a coverage factor *k*. For confidence level of 95% *k* is 2. The expanded uncertainty (at 95% confidence level) was found to be ±12%.

The higher sample weight used in the proposed method (10 g) with accepted solid phase extraction cleanup techniques compared with E1 and E2 QuEChERS acetonitrile based extraction method (1 g) [19] facilitates the ability of lowering the limits of quantification for PAHs where the recoveries obtained at 500 *μ*g/Kg for traditional acetonitrile based QuEChERS extraction using extraction scheme E1 (1% acetic acid in acetonitrile and AOAC salts) yield average recoveries less than 67%, with individual PAHs recoveries typically ranging from 35 to 87%, also for extraction scheme E2 (acetonitrile and EN salts) performed equally poorly, with average PAHs recoveries being less than 68% and individual PAHs recoveries ranging from 24 to 88%, while for the proposed method the individual PAHs recoveries range from 65 to 107% at the LOQ limits (2 *μ*g/Kg) with method uncertainty equal to ±12 (at 95% confidence level) indicating that the method is quite fit for purpose with acceptable LOQ, precision, and accuracy according to Commission Regulation (EC) number 1881/2006 and (EC) number 333/2007.

## 4. Conclusion

The results found were very promising; it may be concluded that modified QuEChERS method of extraction followed by cleanup silica gel packed solid phase extraction combined with GCMS for quantitation is an efficient method for determination of low concentration of selected group of PAHs in homogenized fish samples. This method is suitable for laboratories engaged daily in routine analysis of a large number of samples, and the LOQ of the method is sufficiently attained low in order to be used in the national monitoring program of Egypt for determination of PAHs in fish as well as in imported and exported fish following Codex regulations.

## Figures and Tables

**Table 1:** Representing the PAHs used and respective analytical ions used for quantification.

Compounds name	CAS number	Target compound monitored SIM ions (*m*/*z*)	
		Quant.	Confirm.
Naphthalene	91-20-3	276	277, 274
Acenaphthene	83-32-9	153	154, 152
Acenaphthylene	208-96-8	152	151, 150
Fluorene	86-73-7	166	165, 167
Phenanthrene	85-01-8	178	176, 179
Anthracene	120-12-7	178	176, 179
Fluoranthene	206-44-0	202	203, 200
Pyrene	129-00-0	202	200, 203
Benz(a)anthracene	56-55-3	228	226, 229
Chrysene	218-01-9	228	226, 229
Benzo(b)fluoranthene	205-99-2	252	253, 250
Benzo(k)fluoranthene	207-08-9	252	253, 250
Benzo(a)pyrene	50-32-8	252	253, 250
Indeno(1,2,3-c,d)pyrene	193-39-5	128	127, 129
Dibenzo(a,h)anthracene	53-70-3	278	279, 276
Benzo(g,h,i)perylene	191-24-2	276	277, 274
Pyrene-d_10_	1718-52-1	212	211, 208

**Table 2:** Retention times (RT), regression coefficient (*r* ^2^), regression equation, limit of detection (LOD), and standard deviation (SD) obtained for standards in toluene calibration.

Compounds	RT (min)	*r* ^2^	Regression equation	LOD	SD
(1) Naphthalene	5.241	0.998	*Y* = 2.68*e*4*X* + 2.67*e*5	1.22	0.41
(2) Acenaphthylene	7.815	0.999	*Y* = 1.14*e*0*X* + 1.56*e*2	0.36	0.12
(3) Acenaphthene	8.092	0.999	*Y* = 1.53*e*4*X* + 4.47*e*4	1.17	0.39
(4) Fluorene	9.040	0.999	*Y* = 1.80*e*4*X* + 2.69*e*4	1.64	0.55
(5) Phenanthrene	11.803	0.999	*Y* = 2.48*e*4*X* + 7.34*e*4	0.74	0.25
(6) Anthracene	11.984	0.999	*Y* = 2.23*e*4*X* + 8.11*e*4	1.94	0.65
(7) Fluoranthene	16.077	0.999	*Y* = 2.55*e*4*X* − 3.26*e*4	0.95	0.32
(8) Pyrene	16.720	0.999	*Y* = 2.53*e*4*X* − 3.12*e*4	0.50	0.17
(9) Benzo(a)anthracene	20.285	0.999	*Y* = 8.37*e*1*X* − 9.44*e*2	0.75	0.25
(10) Chrysene	20.409	0.999	*Y* = 9.17*e*1*X* − 7.76*e*3	0.33	0.11
(11) Benzo(b)fluoranthene	23.792	0.998	*Y* = 6.34*e*1*X* − 9.62*e*2	0.57	0.19
(12) Benzo(k)fluoranthene	23.861	0.999	*Y* = 8.40*e*1*X* + 6.26*e*2	0.58	0.19
(13) Benzo(a)pyrene	24.612	0.998	*Y* = 7.78*e*1*X* − 9.25*e*2	0.37	0.12
(14) Indeno(1,2,3-cd)pyrene	27.809	0.996	*Y* = 3.86*e*1*X* − 8.83*e*2	0.44	0.15
(15) Dibenz(a,h)anthracene	27.870	0.996	*Y* = 5.10*e*1*X* − 8.85*e*2	0.90	0.30
(16) Benzo(g,h,i)perylene	28.534	0.999	*Y* = 7.98*e*1*X* − 5.45*e*2	0.09	0.03

**Table 3:** Representing recovery percentage, relative standard deviation (RSD%), and RSD_pooled_% results of *n* = 6 replicates on each spiking level.

Compounds	Recovery ± RSD%			RSD_pooled_%
	2.0 *µ*g/Kg	10.0 *µ*g/Kg	50.0 *µ*g/Kg	
(1) Naphthalene	95 ± 16	96 ± 11	88 ± 5	5
(2) Acenaphthene	96 ± 5	108 ± 10	113 ± 11	5
(3) Acenaphthylene	107 ± 12	118 ± 3	97 ± 5	4
(4) Fluorene	85 ± 10	117 ± 11	114 ± 10	5
(5) Phenanthrene	102 ± 12	109 ± 5	116 ± 10	5
(6) Anthracene	87 ± 10	112 ± 10	115 ± 10	5
(7) Fluoranthene	102 ± 12	106 ± 13	115 ± 9	5
(8) Pyrene	101 ± 13	111 ± 12	115 ± 10	5
(9) Benzo(a)anthracene	96 ± 6	99 ± 4	105 ± 2	2
(10) Chrysene	89 ± 19	88 ± 6	103 ± 2	4
(11) Benzo(b)fluoranthene	76 ± 6	72 ± 4	105 ± 3	3
(12) Benzo(k)fluoranthene	89 ± 10	76 ± 6	97 ± 6	4
(13) Benzo(a)pyrene	70 ± 8	74 ± 13	106 ± 5	4
(14) Indeno(1,2,3-cd)pyrene	65 ± 9	61 ± 7	98 ± 5	4
(15) Dibenzo(a,h)anthracene	74 ± 9	72 ± 9	97 ± 5	4
(16) Benzo(g,h,i)perylene	69 ± 3	56 ± 9	96 ± 5	4
